# Integrating active brain-computer interfaces (aBCIs) with passive BCIs (pBCIs) under different frustration levels

**DOI:** 10.1038/s41598-025-30168-1

**Published:** 2025-12-05

**Authors:** Xin Gao, Haipeng Lin, Xiaolong Wu, Dingguo Zhang

**Affiliations:** 1 Bath Institute for the Augmented Human, University of Bath, Bath, UK; 2 Southern University of Science and Technology, Shenzhen, Guangdong PR China

**Keywords:** Adaptive BCI, Electroencephalography, Active BCI, Passive BCI, Frustration, Engineering, Biomedical engineering

## Abstract

The mental state of the users can significantly affect the performance of active brain-computer interfaces (aBCIs). In this work, we aim to adopt passive BCIs (pBCIs) to measure a typical mental state, frustration, which is much relevant to aBCIs. A novel paradigm has been developed that combines both aBCIs and pBCIs under different frustration levels of users. The aBCI in this work is based on classic binary motor imagery (MI). In experiments, a new strategy was implemented that uses visual feedback to induce different levels of frustration. The electroencephalography (EEG) data collected were used for both aBCIs and pBCIs. The pBCI was utilized to assess the frustration level during the aBCI tasks, and the aBCI classification models for different levels of frustration were trained. For pBCI, the filter bank common spatial pattern (FBCSP) feature extraction and support vector machine (SVM) classification were utilized to classify three (i.e., low, moderate, high) frustration levels. For aBCI, the same method (FBCSP+SVM) was used to classify left versus right MI. We also aim to improve the performance of aBCIs in such conditions, so we developed two new methods to incorporate the pBCI results to adapt three MI classifiers to the varying states of frustration. Compared to the conventional approach of directly classifying MI tasks without considering frustration, the two proposed methods increased the mean classification accuracy by 7.40% and 8.62%, respectively. (Compared with the commonly used non-emotional discrimination data, the results are improved by 4.56% and 5.87% respectively.) Within the scope of non-invasive EEG and MI-based aBCI, this study provides, to our knowledge, an initial integrated demonstration in which a frustration-level classifier (pBCI) is trained and then used to adapt MI decoding (aBCI). It should not be taken as a claim of originality beyond this context. Starting from “user subjective perception”, this paper rises to the engineering level of “objective frustration recognition and classification model adaptation”, and makes a contribution to the depth of EEG data analysis and methodological integrity.

## Introduction

The goal of brain-computer interfaces (BCIs) is to allow the user to communicate without the participation of peripheral nerves and muscles. Based on the level of invasiveness of the signals acquired, BCI can be categorized into invasive, semi-invasive, and non-invasive types^1^. Among them, electroencephalography (EEG) has attracted extensive attention due to its non-invasiveness and high temporal resolution. From the perspective of application scenarios, BCI can be classified into active BCIs (aBCI), which involves control paradigms such as motor imagery (MI), reactive BCIs (rBCI) such as visual evoked steady-state potentials (SSVEP, P300), and passive BCIs (pBCI), which focuses on measuring states without explicit user commands, such as emotional BCIs^2^.

In EEG-based aBCI systems, data are acquired, preprocessed, features are extracted/classified, and control instructions are generated for external devices or graphical user interface (GUIs). The low signal-to-noise ratio of EEG signals limits their application. Leveraging passive states, such as psychological or emotional conditions, is a promising approach to enhancing aBCI system performance, alongside improving signal quality and feature extraction methods^3–5^. The emotional state, fatigue, and cognitive states of the human brain are intricately linked to neural activities. These neural behaviors, not governed by conscious will, are often considered passive or latent brain tasks^6,7^. In the realm of pBCI, explorations have been conducted aimed at more precisely identifying the participant’s mental state. Venkatesh and Yi’s work used the number of simulated driving infractions, blink frequency, and subjective reports to gauge varying levels of fatigue, using a classifier to recognize in the gathered data the fatigue levels of participants^8,9^ . EEG was used to measure user engagement during reading, with EEG data classified under different engagement conditions based on user self-report, and least-squares regression (LSR) thresholds methods were applied to detect engagement status during reading^10^. Related research also encompasses areas such as arousal, vigilance, and working memory load^11–13^. However, while most studies focus on either aBCI or pBCI, relatively few efforts have integrated these two paradigms.

Regarding the adaptability of aBCIs based on different mental states, an approach is to filter the training data set using a different mental state and update the classifier parameters^14,15^. For instance, Andrew and Tom linked self-reported fatigue, attention, and frustration levels with EEG-based aBCI performance and reported that moderate fatigue and medium to high frustration could improve classification precision^3^. However, reliance on delayed self-reports introduces potential biases and limited temporal precision. Another study employing wavelet packet best basis decomposition (WPBBD) have demonstrated improved classification accuracy by tailoring feature extraction to individual subjects^16^. However, these methods assume static mental states during data collection, limiting their ability to address synchronous fluctuations such as fatigue or frustration, and their reliance on fixed wavelet functions further restricts adaptability across diverse tasks or users.

Other studies have tracked user fatigue with more advanced methods, such as kernel partial least squares or adaptive updating of spatial filters^17^, but they often depend on infrequent assessments, reducing responsiveness to fast-changing mental states. Even attempts to alleviate frustration by manipulating task outcomes only partially address the complexity of emotional dynamics^18^.

Instead of rigidly classifying passive states, some approaches dynamically adjusts training datasets or feature capture methods based on user states and environmental conditions. Olivier et al. used amplitude modulation (AM) features to improve the aBCI task accuracy of classification , but this passive feature operates on a temporal scale that may not fully capture rapidly evolving mental states^14^. Similarly, adaptive CSP (ACSP) algorithms, which combine regularization of recursive least squares (RLS) and diagonal loading (DL), dynamically update the CSP filter coefficients to reduce calibration time and improve MI classification^19^. Despite their efficiency, these methods depend on hyperparameters like RLS and DL, which pose challenges for optimization and overlook the impact of dynamic mental states on BCI performance. Meanwhile, shrinkage adaptive projected sub-gradient APSM introduces controllable shrinkage effects and intuitive hyperparameter tuning strategies, enabling adaptability to rapidly changing environments and nonstationary signals^20^. However, its primary focus on time-domain features and reliance on stable theoretical assumptions limits its robustness in diverse and dynamic real-world scenarios.

These approaches highlight critical gaps in existing research. First, most of the studies mentioned explored differences in the efficiency of aBCI in different mental states, and few studies integrated these differences into an aBCI system based on pBCI state discrimination, thus optimizing the aBCI effect in the face of dynamically changing passive states. Second, when collecting mental state data, experiments generally use scales to evaluate the subjects’ accompanying mental states and artificially assign labels to the evaluated data segments^3,14,16^. Reliance on self-report or sparse data annotations constrains the capacity to capture more precise mental states. Lastly, while adaptive BCI methods dynamically adjust training datasets or feature capture methods, they often focus solely on algorithmic or signal processing improvements without effectively leveraging passive mental state information. This limits their ability to address the impact of emotional and cognitive fluctuations on BCI performance. For example, in the work of Myrden and Chau^3^, only the changes in the classification accuracy of aBCI under different emotional states were mentioned, but the pBCI classification and aBCI were not tested and compared together. This limits their ability to address the impact of emotional and cognitive fluctuations on BCI performance.

By incorporating mental state information, such as frustration levels, into active classification methods. Frustration is a critical mental state indicator with significant implications for BCI performance. Inefficient decoding in BCIs often leads to frustration for users when controlling systems through EEG signals^2^. The work of^21^ also illustrates the importance of human emotions in assisting control systems. Furthermore, task complexity within BCI applications significantly influences user experience and frustration levels.

In light of these considerations, this study focuses on frustration, an often overlooked but influential mental factor, to examine its effects on the efficiency of aBCI performance. Frustration, characterized by feelings of annoyance stemming from an inability to achieve a goal^22^, is a critical mental state. Informed by this definition, the experiments were designed to induce frustration. In this work, the researchers let users use MI in a GUI program to control the left and right movements of a small ball. The direction of the ball’s movement is not actually controlled by the machine learning model trained using MI data in the first stage, but by a random number generator designed with different accuracies depending on the desired level of frustration induction. While prior work has manipulated perceived control/frustration and reported its relationship with BCI performance, we are not aware—in the specific context of EEG-based MI—of a study that (i) induces graded frustration probabilistically, (ii) trains a dedicated multi-class pBCI on those data, and (iii) uses the pBCI outputs to adapt MI decoding in the same pipeline. Accordingly, our contribution is a proof-of-concept for adaptive aBCI based on information from pBCI (i..e. frustration). Methodologically, the contribution lies in integrating a graded frustration recognizer (pBCI) with MI decoding (aBCI) via hard switching and probabilistic fusion. We intentionally use FBCSP as a transparent baseline to isolate the value of state-aware adaptation. The framework is model-agnostic and compatible with alternative backbones (e.g., Riemannian geometry^23^, EEGNet/DeepConvNet^24^, transformer-based models) without changing the adaptation layer.

This induction of frustration offers an opportunity to:

- Empirically illustrate the influence of frustration on aBCI: We provide data-driven evidence that controlled induction of frustration, achieved through probabilistic feedback, can significantly affect the performance of aBCI systems, indicating the importance of incorporating such states into system design.
- Characterization of frustration via EEG: By collecting EEG data during precisely manipulated frustration levels, this study contributes to a more immediate and objective assessment of passive mental states.
- Propose integrated aBCI+pBCI adaptation methods: We introduce and evaluate two methods that incorporate frustration-level information into aBCI frameworks, demonstrating that such integration can enhance MI classification performance and offer insights for more adaptive and user-centric BCI designs.

The next sections detail the experimental design and methods employed in this study. We first describe how participants were recruited and how we collected baseline MI data. We then explain the induction of frustration via a controlled probabilistic feedback mechanism and the recording of EEG signals under varying frustration conditions. Following this, we present the methodological approaches used for data processing, model training, and the introduction of two novel aBCI+pBCI fusion methods.

## Results

### Data analysis at different states of frustration

The data of the aBCI process were analyzed at three different levels of frustration. Spectrum, statistical significance test, source localization and aBCI classification results under different levels of frustration were analyzed.

**Fig. 1:**
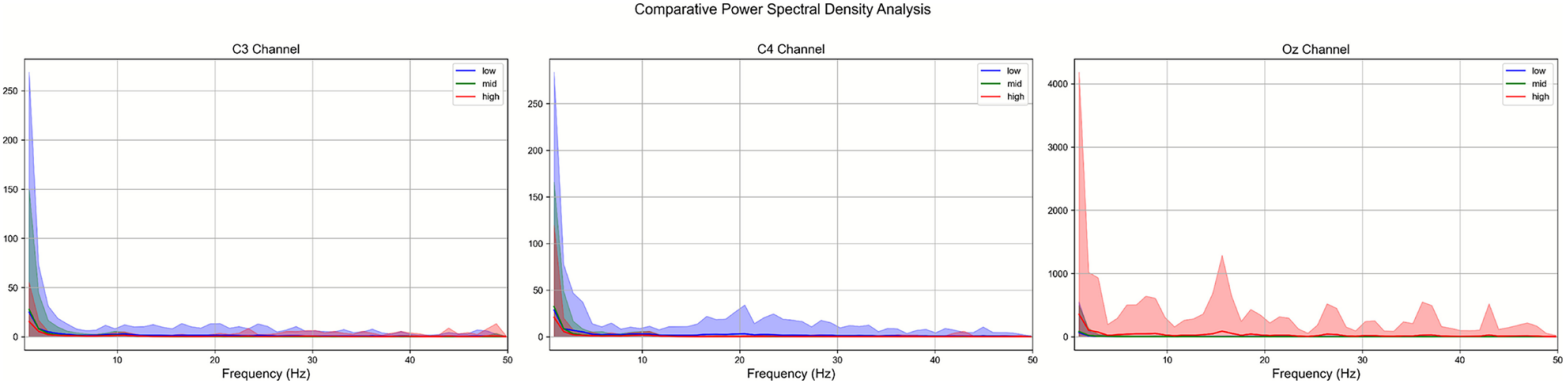
The Evoked spectrum of the three induced frustration states is shown in the red, green and blue lines above, where the x-axis is frequency and the y-axis is power gain in decibels. The shaded part of the statistical line in the figure represents the variance of the state curve. From left to right, it is the C3, C4 and Oz spectrum related to all channels, motor sensory area.

Figure 1 shows a spectrum diagram of all subjects participating in the experiment. From left to right, the figure represents the average spectrum of all participants on C3, C4, and Oz channels. The blue, green, and red lines represent different spectrums under low, medium, and high levels of frustration, respectively. The semi-transparent bands around each line indicate the upper and lower bounds of the standard deviation of the spectrum, showing the variability among the participants.

It can be observed that in the low-frequency range (2-8Hz), the spectrum under low frustration is a bit higher from those under high and medium frustration states. The C3 and C4 channels basically maintain a consistent trend with the mean. The low-frequency activity under low frustration levels is relatively active, but in the Oz brain area of the occipital lobe, the high frustration levels state shows stronger spectral energy in 1-20Hz. Overall, low frustration has the strongest low-frequency response among the three frustration categories. From the spectrogram on the Oz channel, the occipital region may have a more significant response to high frustration.

**Fig. 2:**
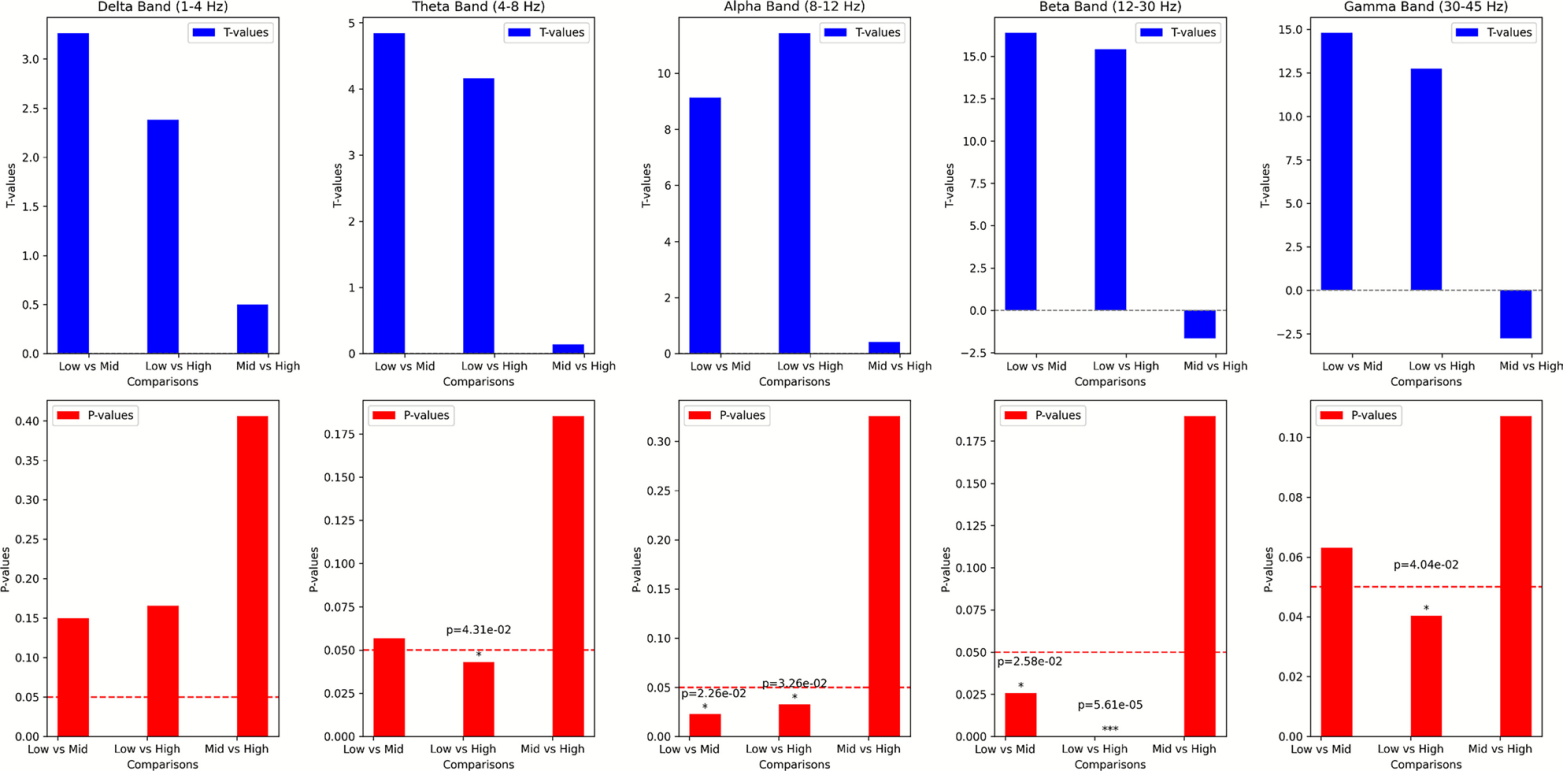
The upper part is the T-value comparison of the PSD mean statistics of all trials under different states, and the lower part is the p-value comparison. From left to right are the Delta, Theta, Alpha, Beta and Gamma frequency bands of the EEG signal. There are three data columns in each image, representing the corresponding t-value and p-value of Low vs mid, Low vs High and mid vs high states respectively.

Figure 2 illustrates the statistical analysis of signal variations across different levels of frustration. It presents the T-values (top row) and p-values (bottom row) for comparisons between Low vs. Mid, Low vs. High, and Mid vs. High frustration levels across five frequency bands: Delta (1–4 Hz), Theta (4–8 Hz), Alpha (8–12 Hz), Beta (12–30 Hz), and Gamma (30–45 Hz). In each frequency band, an independent sample T test was performed on the PSD between different conditions (Low vs Mid, Low vs High, Mid vs High), and the T and p-values were calculated. The p-value and t-value are obtained by calculating the PSD of the aBCI data of the same subject at different levels of frustration. From the perspective of p-values, in the Beta and Gamma bands, Low vs mid and mid vs high have stronger statistical significance. In the Delta, Theta and Alpha bands, although the t-value shows that there are certain differences between different frequency bands and different levels of frustration, the p-value analysis shows that this difference is non-stationary. From the data source, this may be because the data for comparison come from the same subject, and the PSD of its EEG signal has a certain degree of autocorrelation even at different levels of frustration. In the Beta frequency band, the t-value of the significant difference between Low vs mid reached -7.46, and the p-value reached 0.005, indicating that the EEG signals in these two states have significant distinguishable characteristics in the Beta band; in addition, in the Gamma frequency band, the t-value of Low vs Mid is -10.87, the p-value is 2.26e-6, and the t-value of low vs high is -6.32, and the p-value is 0.0087; This shows that the Low vs mid and low vs high states have reliable distinction in this frequency band.

### Subjective validation of frustration induction

To validate the effectiveness of the frustration induction protocol, participants rated their subjective frustration on a 7-point Likert scale immediately after each block. A repeated-measures ANOVA revealed significant overall differences among the three task success-rate conditions (80%, 65%, 50%), $F(2, N-1) = \ldots , p < 0.01$. The mean frustration scores were $M=2.1 \pm 0.8$ in the 80% condition (low), $M=3.5 \pm 1.4$ in the 65% condition (medium), and $M=4.8 \pm 1.2$ in the 50% condition (high), demonstrating a clear stepwise increase in frustration with decreasing task success probability. While a small number of participants (e.g., Subjects 7 and 12) showed minor deviations from the expected order, these individual differences did not affect the overall statistical significance or the monotonic trend. These findings indicate that the manipulation reliably induced the intended gradient of frustration, consistent with theoretical expectations in BCI and psychological research.

### Performance of the pBCI classifier

To directly evaluate the reliability of the pBCI component, we analyzed the classification performance of the frustration-level classifier using a five-fold cross-validation procedure. Table 1 reports the classification accuracy and standard deviation for each of the 12 participants. Across all subjects, the classifier achieved an overall mean accuracy of 85.2% with relatively low variance, demonstrating stable and robust performance. Individual accuracies ranged between 81.3% and 90.2%, suggesting that the classifier was effective across different participants.

**Table 1:** Classification accuracy and standard deviation (%) of the frustration-level (pBCI) classifier across 12 participants. Overall, the classifier achieved a mean accuracy of **85.2%** ± 0.096, demonstrating robust discrimination between low, medium, and high frustration states. As noted in the **Results** section, this relatively high accuracy may partly reflect the temporal autocorrelation of EEG signals, which can increase apparent separability between conditions. Nevertheless, the results support the reliability of the pBCI classifier as a crucial intermediate component of the proposed framework.

	1	2	3	4	5	6	7	8	9	10	11	12	**Avg.**
pBCI Accuracy (%)	84.2	87.5	81.3	83.6	86.7	82.9	88.1	84.5	90.2	83.7	86.1	83.3	**85.2**
pBCI Std.	0.045	0.152	0.061	0.158	0.049	0.072	0.244	0.066	0.037	0.159	0.051	0.063	–

Table 2 further presents the averaged confusion matrix across all participants. The classifier exhibited strong discrimination of low and high frustration states, with recall rates of 88.1% and 85.9% respectively. As expected, the medium condition was more difficult to classify, showing greater overlap with both neighboring states, and yielding a recall of 80.3%. Precision values showed a comparable trend, with medium frustration slightly lower (82.1%) compared to low (86.7%) and high (86.5%) frustration.

**Table 2:** Confusion matrix of the frustration-level (pBCI) classifier averaged across all participants. Overall classification accuracy was **85.2%**. The classifier shows robust discrimination of low and high frustration states, while medium frustration exhibits more overlap with both extremes. The relatively high accuracy may partly reflect EEG temporal autocorrelation, which increases apparent separability between conditions.

True / Predicted	Low frustration	Medium frustration	High frustration	Recall (%)
Low frustration	88.1%	7.9%	4.0%	88.1
Medium frustration	9.8%	80.3%	9.9%	80.3
High frustration	3.7%	10.4%	85.9%	85.9
**Precision (%)**	86.7	82.1	86.5	–

Importantly, the relatively high overall accuracy may partly reflect the temporal autocorrelation inherent in EEG signals, which can increase apparent separability between conditions when adjacent trial segments are not fully independent. Nevertheless, even considering this factor, the results confirm that the pBCI classifier provides sufficiently reliable estimates of participants’ frustration states, thereby validating its role as a crucial intermediate component of the proposed framework and supporting its contribution to downstream aBCI performance improvement.

### Training results of pBCI+aBCI

In the decoding performance analysis of aBCI, the MI classification results under three frustration levels are shown in Table 3, among which the model specialized for low frustration level aBCI data achieved the highest classification accuracy of 76.94% in three different modes, with a higher variance. The average accuracy of MI under medium and high frustration was 72.92% and 61.57%, respectively. The average discrimination accuracy of the mean model is 62.75%, which is lower than the discrimination model trained separately under the three frustration levels. Among the 12 subjects, two had MI accuracy under low frustration reaching 90%. From the results of the accuracy Wilcoxon signed rank test, the p-value of the low frustration state was 0.0089 and 0.021 compared to the medium and high group, showing a significant difference.

**Table 3:** MI classification accuracy and standard deviation (%) for each of the 12 subjects across three frustration-specific conditions (Low, Medium, High) and a state-agnostic baseline condition. Boldface indicates, for each subject, the highest accuracy among the three frustration-specific conditions (Low/Medium/High). The *All States (Mean)* condition corresponds to a state-agnostic baseline, where data and labels from all three frustration states are combined for training and testing, without considering differences in pBCI (frustration) levels.

	1	2	3	4	5	6	7	8	9	10	11	12
Low Frustration Accuracy (%)	**85.01**	91.02	**72.99**	70.04	**87.44**	**68.18**	**87.12**	57.73	**97.55**	78.72	**74.33**	53.20
Low Frustration Std.	0.072	0.030	0.082	0.012	0.030	0.230	0.120	0.150	0.070	0.090	0.030	0.220
Medium Frustration Accuracy (%)	70.00	**95.03**	67.67	**74.25**	78.65	60.33	76.28	60.09	91.00	74.17	67.43	60.10
Medium Frustration Std.	0.065	0.040	0.130	0.060	0.030	0.220	0.140	0.092	0.210	0.174	0.090	0.130
High Frustration Accuracy (%)	55.21	67.69	59.23	55.27	53.25	51.24	64.65	64.40	73.20	**83.36**	46.71	**64.69**
High Frustration Std.	0.150	0.230	0.170	0.220	0.310	0.110	0.080	0.140	0.270	0.110	0.160	0.220
All States (Mean) Accuracy (%)	63.17	83.25	54.41	58.75	64.03	47.17	63.70	**65.81**	74.49	56.93	66.67	54.73
All States (Mean) Std.	0.220	0.330	0.120	0.130	0.090	0.140	0.173	0.182	0.224	0.120	0.230	0.320

**Fig. 3:**
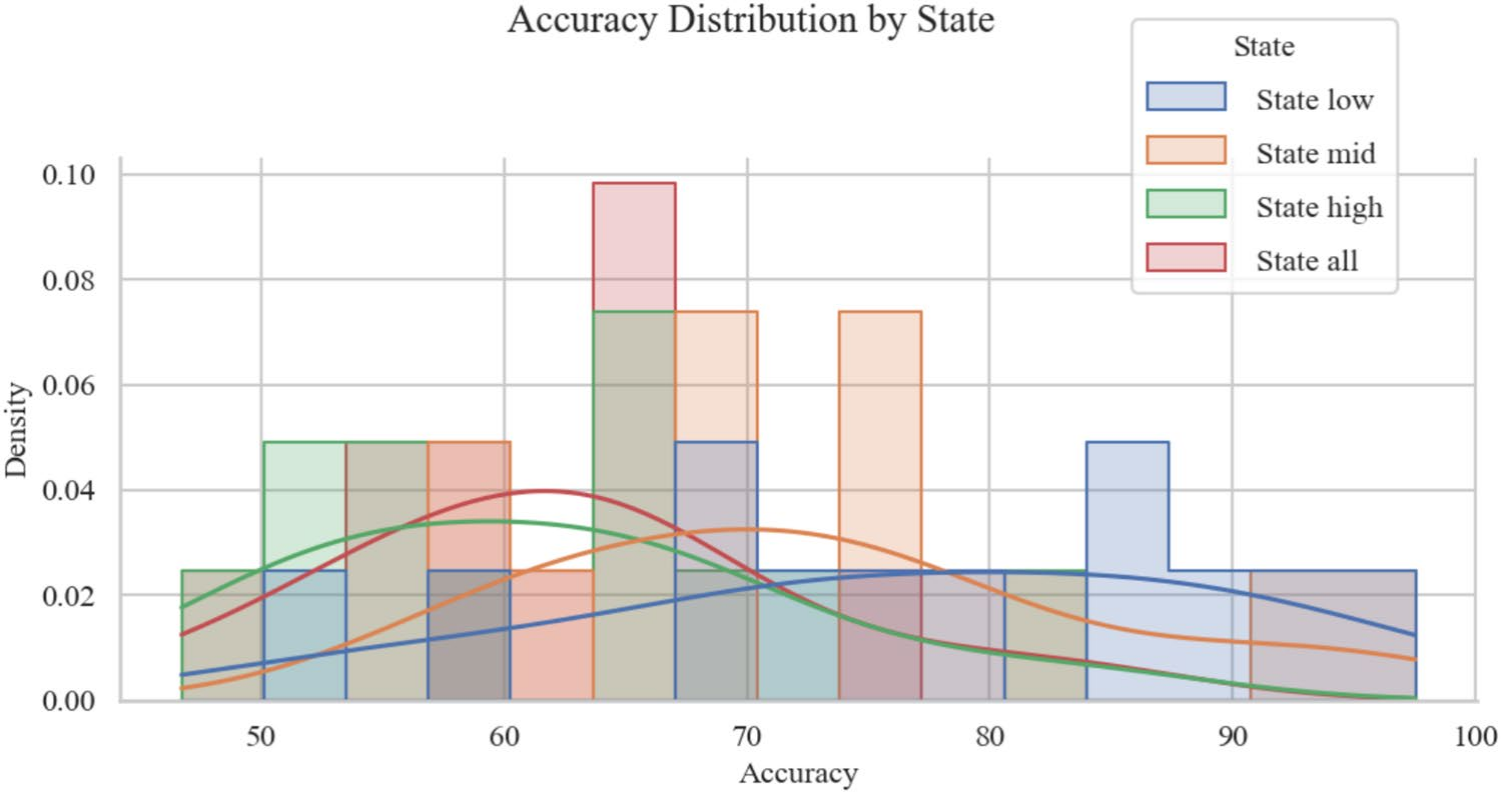
Accuracy distribution for motor imagery (MI) classification under three frustration-specific conditions (*State low*, *State mid*, *State high*), as well as a *State-all (All data in low, mid and high stage)* condition. Histograms and Kernel Density Estimation(KDE) curves illustrate performance variability across subjects. The state-all baseline corresponds to a conventional classifier trained and tested on the combined dataset of all three frustration states, without considering differences in pBCI (frustration) levels.

In addition to the statistical comparison, the distribution of MI classification accuracy across different frustration states is visualized in Fig. 3. The figure shows both histograms and kernel density estimates for each condition. It is evident that accuracy under low frustration (*State low*) is generally skewed toward higher values, with a heavier tail approaching 90%. In contrast, the distribution under high frustration (*State high*) is wider and centers around lower accuracy values, indicating reduced and more variable MI decoding performance. The medium frustration state lies between the two, while the combined condition (*State all*) smooths out individual differences and yields intermediate performance. This visual analysis further confirms that frustration state has a clear impact on MI-based classification, reinforcing the need for frustration-aware model adaptation in aBCI systems.

**Fig. 4:**
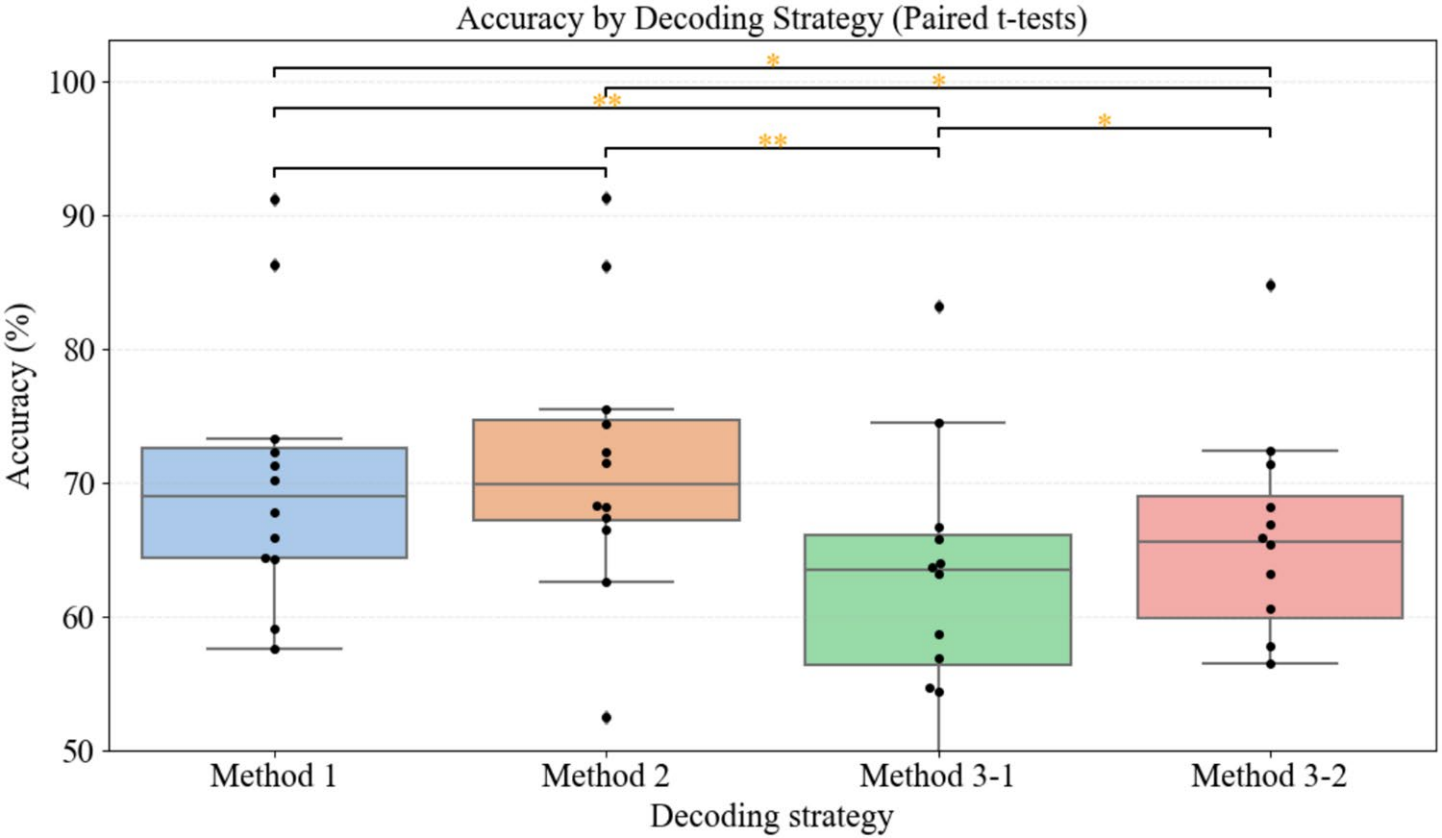
Distribution of motor imagery (MI) classification accuracies across four decoding strategies in the aBCI system: Method 1 (frustration-state classifier followed by state-specific MI models), Method 2 (probabilistic fusion of frustration-state and MI classifiers), Method 3-1 (conventional state-agnostic MI decoder), and Method 3-2 (baseline MI decoder trained on non-frustration data). Each boxplot summarizes results from 12 participants, with individual subject accuracies overlaid as black dots. Horizontal brackets indicate paired comparisons between methods, with statistical significance based on paired t-tests. Orange asterisks plotted directly on the brackets denote the level of significance (* p < 0.05, ** p < 0.01, *** p < 0.001).

As shown in Fig. 4 and Table 4, Method 1 yielded an average MI classification accuracy across all participants of 70.15%, which represents a 7.40% improvement over the state-agnostic baseline (Method 3-1, 62.75%) and a 4.56% improvement over the initial non-frustration baseline (Method 3-2, 65.59%).

**Table 4:** Classification accuracy and standard deviation (%) of motor imagery (MI) decoding across 12 participants using four different decoding strategies described in the **Methods** section. **Method 1** incorporates a frustration-state classifier followed by state-specific MI models; **Method 2** uses probabilistic fusion of frustration-state and MI classifiers; **Method 3-1** applies a conventional, state-agnostic MI decoder; **Method 3-2** represents the initial non-frustration MI data (from the first stage). Bold values denote the highest accuracy achieved per subject across the methods.

	1	2	3	4	5	6	7	8	9	10	11	12	Avg.
Method 1 Accuracy (%)	67.74	**86.27**	65.89	70.21	**72.31**	57.55	71.23	64.33	91.22	73.30	64.32	**59.11**	72.15
Method 1 Std.	0.072	0.670	0.030	0.082	0.012	0.130	0.230	0.120	0.020	0.060	0.040	0.210	0.142
Method 2 Accuracy (%)	**71.50**	86.22	**66.48**	**72.23**	68.23	**62.58**	**74.33**	67.36	**91.24**	**75.52**	**68.22**	52.50	**71.37**
Method 2 Std.	0.061	0.055	0.062	0.094	0.073	0.087	0.097	0.150	0.030	0.055	0.057	0.077	0.075
Method 3-1 Accuracy (%)	63.21	83.22	54.39	58.68	64.00	47.17	63.71	65.82	74.51	56.88	66.72	54.71	63.68
Method 3-1 Std.	0.073	0.140	0.150	0.140	0.150	0.240	0.130	0.040	0.180	0.120	0.080	0.130	0.131
Method 3-2 Accuracy (%)	65.36	84.73	56.51	63.22	65.83	43.47	66.83	**71.36**	72.37	60.58	68.19	57.81	65.9
Method 3-2 Std.	0.051	0.062	0.120	0.230	0.068	0.252	0.234	0.079	0.237	0.103	0.093	0.170	0.141

Method 2, which introduces a more refined probabilistic fusion of state and MI classifier outputs, delivered the highest average accuracy of 71.37%, along with a lower inter-subject variance of 0.075. Compared to Method 3-1, Method 2 improved accuracy by 8.62%, and compared to Method 3-2 it improved accuracy by 5.78%. Moreover, Method 2 demonstrated greater consistency, with four participants exceeding 75% accuracy—highlighting its practical reliability in real-world applications.

In contrast, Method 3-1, which does not incorporate any affective state modeling and mixes all frustration-level data for training, yielded the lowest average classification accuracy (62.75%) and the highest inter-subject variance (0.131). Method 3-2, which trained only on the initial non-frustration MI data, achieved a slightly higher accuracy (65.59%) and smaller variance (0.097) than Method 3-1, but still underperformed compared with the adaptive approaches.

Statistical analysis using paired t-tests revealed that both Method 1 and Method 2 significantly outperformed Method 3-1 and Method 3-2 (p < 0.01). Although the average performance of Method 2 was slightly higher than that of Method 1, the difference did not reach statistical significance. Both methods share the same preprocessing and classifier training pipeline, differing only in their final decision strategies: Method 1 applies hard classification based on the most probable state, while Method 2 applies soft probabilistic weighting across classifiers. These differences appear to affect stability more than absolute accuracy, suggesting that Method 2 may offer better robustness—a valuable trait for online deployment.

## Discussion

### Source localization across frustration levels

**Fig. 5:**
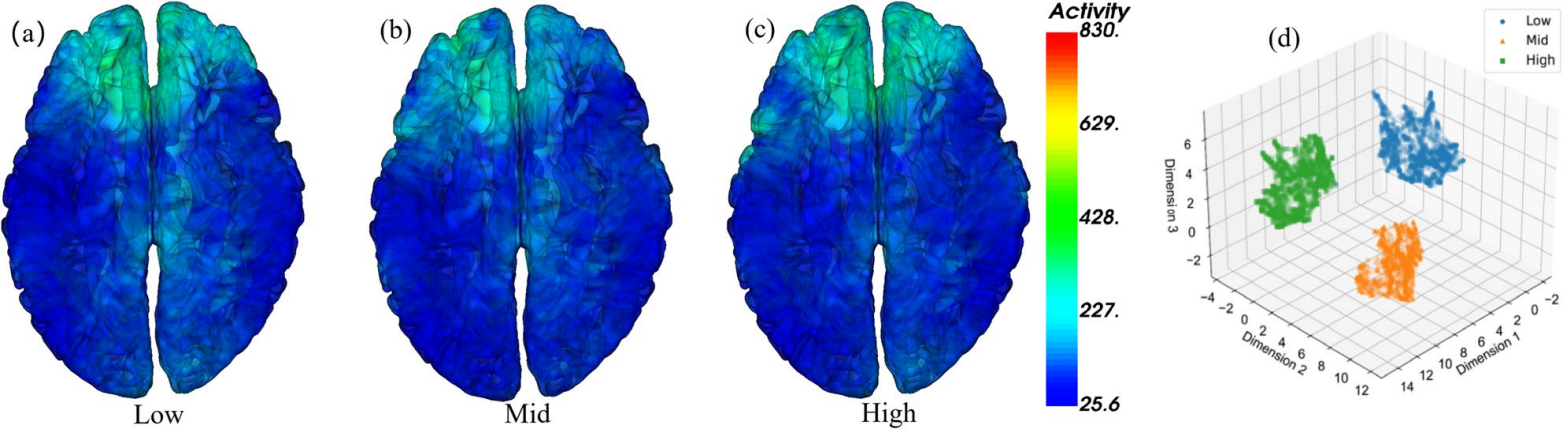
Brain source localization and feature separability under different frustration levels. Panels (**a**), (**b**), and (**c**) show sLORETA-based brain source localization results under low, medium, and high frustration conditions, respectively. The visualization is based on group-averaged aBCI data recorded from 0.5 to 2.5 seconds post-cue during motor imagery tasks, mapped using the HCP standard head model. Stronger activation in motor and occipital areas is observed in the low-frustration state. Panel (**d**) shows UMAP-based dimensionality reduction of the EEG features, where samples from low (blue), medium (orange), and high (green) frustration levels form clearly separated clusters. This indicates high inter-class separability of neural representations across emotional states.

The a, b, and c sub-graphs shown in Fig. 5 are the results of brain source localization under different levels of frustration. The method of brain source localization is based on sLORETA. The head model data in the lead field for the location of the brain source uses standard HCP data^25^, which are segmented by Charm^26^. The target mapping brain source uses the grid data and the front-lead field of the standard head model. All epochs of all subjects are grouped under different levels of frustration and all aBCI data are averaged. The aBCI data uses the data from 0.5 seconds to 2.5 seconds after the ball starts to move in Experiment 2 for brain source localization. In the actual drawing, we average the time series data. It can be seen from the figure that the average brain source activity under low frustration is stronger than that under medium and high frustration. Compared with the low frustration state, the motor sensory area activity in the medium and high frustration state is weaker. Both high and low frustration show a certain degree of occipital lobe activity. In contrast, the occipital lobe activity in the medium frustration is the lowest, which may be related to the sense of perceived control and the trust in the brain control system. According to Brodmann’s partitioning^27^, the primary motor cortex and the superior parietal lobule show a certain trend of decreasing source activity from low frustration to high frustration, which may be related to the emotional activity of the brain area and is consistent with the weakening of Frontal Midline activity under negative emotions mentioned in the^28^ study. The EEG signals used in this study for statistical analysis of brain source localization were the average data of all subjects during active motor imagery under different frustration states. Therefore, the differences in brain source activity presented in the figure are more likely to reflect macro differences at the group level rather than direct manifestations of minor differences between individuals.

### Feature separability (UMAP)

The results of the uniform manifold approximation and projection (UMAP) visualization demonstrate that, after dimensionality reduction, the brain activity data corresponding to low frustration (blue dots), medium frustration (orange triangles) and high frustration (green squares) exhibit clear clustering and spatial separation. This indicates that the neural representations of different frustration levels occupy distinct regions in the high-dimensional feature space. Quantitatively, the average Euclidean distance between cluster centroids of low and high frustration levels was greater (e.g., $d_{\text {low-high}} = 5.72$) compared to that between medium and high frustration levels ($d_{\text {mid-high}} = 3.46$), suggesting a higher separability between the two extremes. Additionally, the inter-class variance to intra-class variance ratio (Fisher’s Discriminant Ratio) exceeded 1.8, further validating the discriminative power of the extracted brain features. A simple linear classifier trained on high-dimensional features achieved accuracy of 95% (cross-validated) to distinguish between low and high frustration conditions, while the classification between medium and high frustration was relatively lower ($\sim$80%). These results not only visually but also statistically support the effectiveness of the frustration induction protocol and demonstrate the reliability of the brain signal patterns associated with distinct frustration states.

Under different levels of frustration, the experimental results suggest that subjects with low frustration seem to be able to obtain more easily distinguishable EEG signals when performing MI. This may be related to low-frequency activities related to emotions, which is consistent with the changes in theta band activity of the hippocampus, anterior cingulate gyrus and prefrontal cortex related to brain emotions mentioned in^29^’s study. Similar studies have also pointed out that frustration usually affects the activities of subjects in relevant frequency bands and corresponding brain areas^30–32^. However, only under low frustration, it seems to show stronger signal steady-state differences. The steady-state difference of the signal is considered to be the basis for providing higher ITR for BCI systems. By analyzing the characteristics of steady-state signals, different brain states, emotional states or other physiological states can be effectively distinguished. More recognizable signals help the neural network model build a clearer feature projection boundary for classification. The studies of^33–35^ have shown a certain theoretical support. The activity of the theta waves of subjects in the hippocampus will increase with positive emotions, and the happy reward-related activities of the gamma wave of the nucleus accumbens and amygdala are reflected in the study by^36^. Positive emotions and low frustration shown in the research of^37,38^ all reflect activities related to the enhancement of neuroplasticity in the dopamine system, hippocampus and prefrontal cortex. These support based on existing physiological conclusions and experimental results may have good potential in the field of external device control supported by EEG. However, unlike^3^, subjects in high frustration may induce higher concentration due to task failure. In the results of my experiment, the average accuracy of the subjects in high frustration was lower than that in medium frustration, which may be related to the means of inducing frustration of the subjects, and in the MI process, the MI signal of the subjects will be mixed with some Errp-related factors.

### Effects of personality

After the collection phase, we administered a questionnaire to characterize personal traits and explore potential associations with outcomes. Seven participants were classified as medium–low resilience, and five (Subjects 1, 5, 8, 10, 12) as high resilience. Descriptively, the three participants whose MI classification was highest under high-frustration conditions (Subjects 8, 10, 12) were all in the high-resilience group; for example, Subject 10 reached 83.36% under high frustration versus 78.72% under low, and Subjects 8 and 12 showed 66.40% vs. 57.73% and 64.69% vs. 53.20%, respectively. This pattern was not observed among lower-resilience participants. Given the small subgroup and inter-individual variability, these observations should be regarded as exploratory. Brief post-experiment notes were broadly consistent with this tendency (e.g., occasional reports of reduced confidence versus maintained engagement), but these qualitative impressions were not analyzed inferentially and are included only to contextualize the descriptive results.

### limitations and outlook

While the present findings demonstrate the feasibility of integrating pBCI and aBCI for frustration-aware adaptation, it is important to emphasize that the current work was conducted in an offline analysis framework. Online deployment introduces several additional challenges, including computational delay in signal processing, the need for robust real-time artifact handling, and the continuous and dynamic nature of affective states that may fluctuate within a session. To move toward practical online applications, future research should focus on optimizing computational efficiency through lightweight feature extraction and classification methods, developing adaptive artifact rejection algorithms that operate in real time, and incorporating temporal models capable of tracking dynamic changes in frustration. In addition, hybrid strategies that combine EEG with complementary physiological signals (e.g., heart rate variability, galvanic skin response) may enhance robustness in naturalistic environments. Addressing these challenges will be essential to translate the offline proof-of-concept presented here into a reliable and scalable online adaptive BCI framework.

This study’s generalizability is constrained by its small sample size (N = 12) and the limited high-resilience subgroup (N = 5), which reduce statistical power and increase uncertainty around effect estimates for the personality analyses. As a result, the apparent link between resilience and MI performance under frustration should be considered preliminary. Future work will address this limitation through adequately powered studies (a priori sample-size planning), pre-registered analyses, and multi-site recruitment to better capture inter-individual variability and enable confirmatory inference. The gains reported here stem from the integration of frustration estimation with MI decoding through hard-switching and probabilistic fusion, not from a novel CSP variant. Future work will benchmark the same adaptation layer with alternative feature/classifier backbones (e.g., Riemannian geometry or deep models) to test generality.

In addition to adaptive BCIs, our joint aBCI–pBCI framework is intended for application domains in which the operator’s mental state materially affects performance. Two concrete targets are: (i) motor rehabilitation based on motor-imagery training, where frustration is common and state-aware adaptation can sustain engagement and improve training efficiency; and (ii) shared-control for human-robot interactions and human-machine systems (e.g., robotic arms, wheelchairs, exoskeletons), where the controller can adjust its level of autonomy according to the estimated frustration state. In these scenarios, the pBCI provides a continuous estimate of frustration that the system uses to modulate assistance weights, task difficulty, and feedback timing—for example, temporarily increasing autonomy or providing corrective cues when frustration rises, and handing back control under low frustration. This state-contingent arbitration aligns with established shared-control principles and offers a practical pathway to deployment^39–41^.

## Conclusion

In this study, we introduced an innovative approach that integrates aBCI and pBCI to enhance MI based BCI performance under varying levels of frustration. By employing synchronous feedback to induce frustration levels and utilizing the FBCSP method, we achieved some improvements in MI classification accuracy. The proposed two methods demonstrated an increase in discrimination accuracy by 7.52% and 8.62%, respectively (Compared with the commonly used non-emotional discrimination data, the results are improved by 4.56% and 5.87% respectively). Our findings highlight the importance of considering mental states in BCI applications, showing that adaptive BCI systems can greatly benefit from real-time assessment of user states. This work not only advances the understanding of frustration’s impact on MI-BCIs but also lays a foundation for the development of adaptive BCIs and shared control. Future research should focus on refining these techniques and exploring their application in real-world BCI scenarios to further validate and extend their efficacy.

## Methods

### Subject recruitment

Twelve healthy participants, comprising eight males and four females with an average age of 26.15 years, completed the two stages of data collection and testing tasks. In the first stage, aBCI training data was collected for left- and right-hand motor imagery (MI). In the second stage, participants’ EEG signals were recorded while they controlled a GUI program under induced states of varying frustration. All participants had normal or corrected-to-normal vision and abstained from caffeine for four hours prior to each data collection session. After the EEG experiment, participants completed the Resilience Scale to explore the relationship between aBCI performance and personality traits, particularly in relation to frustration levels. The scale consists of 25 questions, each rated on a 7-point Likert scale ranging from “strongly disagree” to “strongly agree.” The final results will be accumulated, and those with scores of 146-175 will be classified as high resilience, and those below 145 will be classified as general resilience (including medium resilience and low resilience)^42^. Written Informed consent was obtained from each participant, and the experiment was conducted in compliance with the Declaration of Helsinki^43^. The experimental protocol and data collection process involved in this study were approved by the Health Ethics Committee (REACH) of the University of Bath, and the REACH reference number is EP 20/21 028.

### Data collection for MI-based aBCI

The aBCI data were collected based on the paradigm of left and right-hand MI. During the data collection process, subjects were instructed to sit comfortably in a chair within a noise-isolated laboratory with stable lighting. They were equipped with the Neuracle v3.0 64-channel wireless EEG acquisition system for data collection, the sampling rate was set as 1000 Hz, following the 10-20 standard.

Participants were positioned in front of a computer screen displaying a GUI configured for data collection. Once the data collection program was initiated, text prompts on the GUI would instruct participants to perform MI tasks involving either the left or right hand randomly. The experiment consisted of 100 trials for left-hand MI and 100 trials for right-hand MI, totaling 200 trials. After completing 100 MI tasks, participants were allowed a 10-minute rest period. Each time the subject completes a MI task, the GUI program will actively prompt the subject to rest for six seconds and will provide a countdown for the rest period.

### Data collection for frustration-induced pBCI

Before data collection commenced, participants were informed that the aBCI MI data collected in the first stage would be used to move a ball to the left or right on the GUI interface. During the experiment, participants were instructed to continue executing the MI-driven ball movement in the specified direction.

When the goal was to induce a low frustration state, the success rate for the participant ball movement control task was established at 80%; for a medium frustration state, the success rate was set at 65%; and for a high frustration state, the success rate was set at 50%. Regarding task completion levels and induction, we based our analysis on the literature surveyed, where a task completion rate below 50% is considered below the random level and a task completion rate above 70% is often a threshold for the perceived effectiveness of BCI users^44–46^. Similar thresholds have also been discussed in cognitive psychology research, where the perception of control and task success rate are strongly associated with affective states such as frustration and helplessness^47,48^. Therefore, the chosen accuracy levels (80%, 65%, 50%) are consistent with both BCI-related and psychological findings, supporting their validity as proxies for low, medium, and high frustration levels. .

When the goal was to induce a low frustration state, the success rate for the participant ball movement control task was established at 80%; for a medium frustration state, the success rate was set at 65%; and for a high frustration state, the success rate was set at 50%. Regarding task completion levels and induction, we based our analysis on the literature surveyed, where a task completion rate below 50% is considered below the random level and a task completion rate above 70% is often a threshold for the perceived effectiveness of BCI users^44–46^. Here, we adopt the settings in^45^. This design aligns with the semantic definition of frustration and, compared to the scale filled out by users after the experiment, this frustration-inducing design maintains consistency between the execution of aBCI tasks and the status of pBCI. To further validate the effectiveness of this frustration induction protocol, immediately after completing each block under a specific success rate condition, participants were asked to report their subjective frustration level on a 7-point Likert scale (1 = “not frustrated at all” to 7 = “extremely frustrated”). These subjective ratings were collected to directly assess participants’ internal frustration states and to confirm the correspondence between manipulated task success probabilities and perceived frustration levels.

The order in which each frustration level appeared was randomized across participants in the experiment to ensure that the results were not influenced by the order of the frustration-inducing patterns.

### GUI design

To facilitate signal collection in the first and second stages, we developed a GUI program to guide participants through the entire experimental process. Upon pressing any key on the keyboard, the data collection task is initiated, and prompts for left and right-hand MI appear in the center of the screen in text form and in random order. Participants are required to perform MI for approximately four seconds during the text prompt period, followed by a rest period of about seven seconds after each MI. The program automatically exits upon completion of all acquisition trials.

**Fig. 6:**
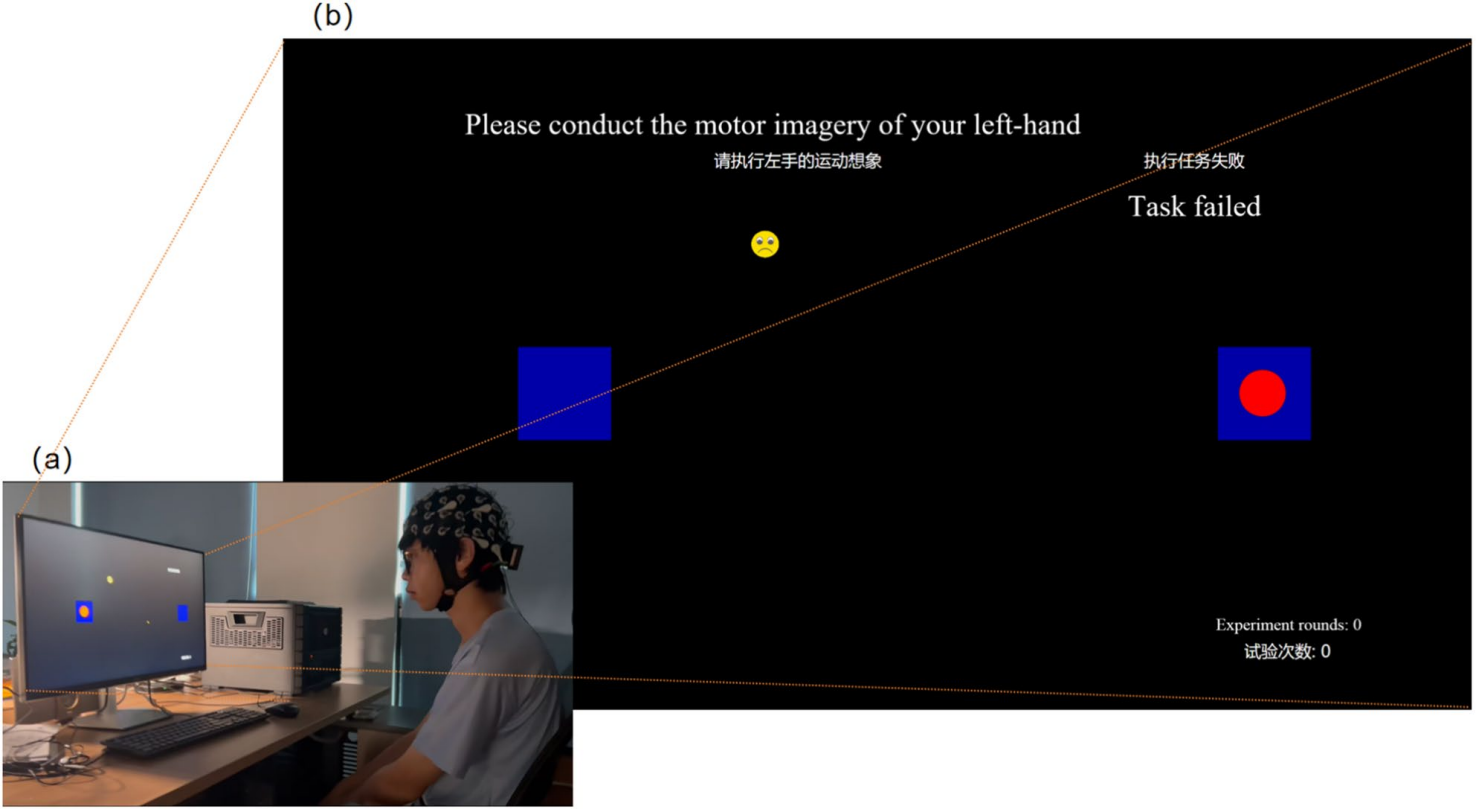
As shown in (**a**), the subject sat in front of a chair and used the MI paradigm to simulate controlling a ball on a GUI, inducing different levels of frustration by the accuracy of the background control. (**b**) is a more detailed example of the GUI, there are two blue square areas on the left and right sides of the screen in front of the user. When the user imagines the movement, the red ball in the picture will move to the left or right with different success rates according to the design of the experiment. When the ball moves to the middle of the blue square area, the screen will prompt success or failure, accompanied by certain sound effects. The lower right corner is the round of the experiment.

In the second phase of the experiment, participants interact with the GUI program depicted in the Fig. 6 to perform MI control tasks. At the onset of this phase, a prompt appears at the center of the screen, instructing participants to engage in a specified MI task for approximately five seconds. Upon completion of this interval, a GUI interface controlling the movement of a ball is presented on the screen. This interface displays two blue square target areas on the left and right sides, with a red ball positioned at the center of the screen. Participants are informed that the movement of the ball will be controlled by their MI. However, the actual movement of the ball is determined by a random probability value, which is configured in the background according to the level of frustration the system aims to induce. The consistency between the ball’s movement and the participants’ intended MI direction is probabilistically controlled; specifically, under conditions designed to elicit higher levels of frustration, the probability of consistency is reduced, whereas under conditions designed to elicit lower levels of frustration, the probability is increased. Once the ball reaches a target area, textual, visual, and auditory prompts provide feedback indicating success or failure.

### Data processing and model training

To investigate the efficiency of MI execution at different levels of frustration, we employed three methods for model training and compared the results. Before the data are fed into the model, it is processed by the pipeline recommended in^49^, the order of preprocessing is baseline correction, 50Hz notch filtering, and 1-40Hz bandpass filtering. The core difference between the three methods mentioned below is that, as shown in Fig. 2 and Fig. 3, both Method 1 and Method 2 are models trained based on data under different levels of frustration, but when finally classifying, Method 1 only considers the classifier identification results under the frustration category with the highest probability, but Method 2 will comprehensively consider the identification results of all classifiers and integrate the probability weights of different frustration levels to weight the MI discrimination results, and finally obtain the MI category after global measurement. The main difference between Method 3 and Method 1 and Method 2 is that Method 3 does not consider the frustration category and uses MI data under all frustration levels for data segmentation, training, and prediction. In the Results section, we used two sets of data to perform calculations and analyses for Method 3. They used data from all levels of frustration( Method 3-1) for training and data from the first text prompt phase( Method 3-2) for training, and their results were called Method 3-1 and Method 3-2, respectively.

**Fig. 7:**
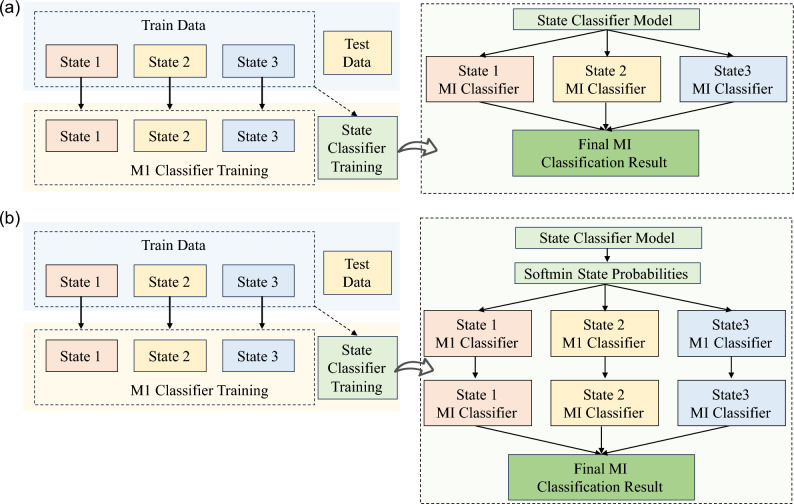
Workflows for two MI classification methods. (**a**) The Hard-Switching method uses a discrete state to select one MI classifier. (**b**) The Probabilistic Fusion method uses a state probability distribution to weight the outputs of all three MI classifiers for a combined final decision.

As shown in Fig. 7(a), Method 1 involved two stages of FBCSP-based modeling.

In the first stage, an affective state classification model was constructed using FBCSP features extracted from EEG segments that had been labeled according to the corresponding frustration levels: namely, level 0 (low), level 1 (medium), and level 2 (high). This frustration-level classification model was trained to distinguish the user’s current emotional state during aBCI interaction and served as the foundation for downstream decision making. During testing Eq. (1) - Eq. (2), an input EEG sample $\textbf{X} \in \mathbb {R}^{C \times T}$ is first processed by the frustration classifier to produce a probability vector:

1 $$\begin{aligned} \textbf{p}_f = [p_0, p_1, p_2], \quad \text {where} \quad \sum _{i=0}^{2} p_i = 1. \end{aligned}$$

Only the frustration category with the highest probability is used to select the MI model:

2 $$\begin{aligned} \hat{s} = \arg \max (\textbf{p}_f), \quad \hat{y} = \arg \max \left( \mathcal {M}_{\hat{s}}(\textbf{X}) \right) . \end{aligned}$$

where $\textbf{X} \in \mathbb {R}^{C \times T}$ denotes the input EEG segment, where *C* is the number of channels and *T* is the number of time samples. The vector $\textbf{p}f = [p_0, p_1, p_2]$ represents the output probabilities from the frustration classifier, corresponding to the likelihoods of low, medium, and high frustration levels respectively, with $\sum {i=0}^{2} p_i = 1$. The index $\hat{s} = \arg \max (\textbf{p}f)$ identifies the most probable frustration level. Based on this, the corresponding motor imagery classifier $\mathcal {M}{\hat{s}}$ is selected, and the final MI prediction is given by $\hat{y} = \arg \max \left( \mathcal {M}_{\hat{s}}(\textbf{X}) \right)$, where $\hat{y}$ indicates the predicted MI class (e.g., left or right hand).

In the second stage, three separate motor imagery (MI) classification models were developed, one for each frustration level. Each of these models was independently trained using EEG data filtered by the corresponding emotional label, and each model specifically targeted the classification of left- versus right-hand motor imagery tasks. Like the frustration model, the MI classifiers also utilized FBCSP for feature extraction, followed by linear discriminant analysis (LDA) or support vector machine (SVM) for classification, depending on the subject’s calibration performanceWe use FBCSP for its robustness and interpretability in MI. Our goal is to evaluate how state-aware adaptation (hard selection vs. probabilistic fusion) affects MI decoding, rather than to introduce a new feature extractor. The pipeline is model-agnostic: any MI classifier (e.g., Riemannian, shallow/deep CNNs, transformers) can be plugged in per state, while the adaptation layer remains unchanged.

During testing, data first passed through the state classifier to determine the data’s frustration level category. EEG data was then sent to the MI classifier specifically trained for that category, resulting in the final determination of the MI category.

As shown in Fig. 7(b), Method 2 introduces a probabilistic fusion strategy to improve MI classification by fully utilizing the uncertainty in frustration state predictions. In contrast to Method 1, which uses a hard decision rule based on the most likely emotional state, Method 2 leverages the entire probability distribution output of the frustration classifier.

In the training phase, FBCSP is applied to extract features from EEG signals labeled by frustration levels (low, medium, high). Based on these features, three frustration-specific MI classifiers $\mathcal {M}_0$, $\mathcal {M}_1$, and $\mathcal {M}_2$ are independently trained to classify left versus right hand motor imagery.

During testing, each EEG input $\textbf{X}$ first passes through a frustration level classifier, producing a probability vector $\textbf{p}_f = [p_0, p_1, p_2]$ over the three emotional states. Rather than selecting the state with the highest probability (as in Method 1), Method 2 computes the MI predictions of the three classifiers. Like Eq. (3) - Eq. (5):

3 $$\begin{aligned} \textbf{p}_i^{\text {MI}} = \mathcal {M}_i(\textbf{X}) = [q_{i,\text {left}}, q_{i,\text {right}}], \quad i = 0,1,2. \end{aligned}$$

These outputs form a $3 \times 2$ tensor, which is then fused using the state probabilities.

4 $$\begin{aligned} \textbf{p}_{\text {final}}^{\text {MI}} = \sum _{i=0}^{2} p_i \cdot \textbf{p}_i^{\text {MI}}. \end{aligned}$$

The final MI prediction is obtained by:

5 $$\begin{aligned} \hat{y} = \arg \max \left( \textbf{p}_{\text {final}}^{\text {MI}} \right) . \end{aligned}$$

where $\textbf{X}$ is the input EEG sample, and $\textbf{p}_f = [p_0, p_1, p_2]$ is the probability vector output by the frustration-level classifier, where $p_0$, $p_1$, and $p_2$ correspond to the predicted probabilities of low, medium, and high frustration levels, respectively. For each emotional state $i \in \{0,1,2\}$, $\mathcal {M}_i(\textbf{X}) = \textbf{p}_i^{\text {MI}} = [q_{i,\text {left}}, q_{i,\text {right}}]$ denotes the output of the corresponding MI classifier, giving the predicted probabilities for left- and right-hand motor imagery. These three outputs are weighted by the state probabilities $p_i$ and summed to produce the final fused probability vector $\textbf{p}_{\text {final}}^{\text {MI}}$. The final MI decision $\hat{y}$ is the class (left or right) with the highest probability in this fused output.

This soft decision approach mitigates errors from ambiguous emotional states. For example, if the frustration classifier outputs [0.40, 0.38, 0.22], Method 1 would commit fully to the “low” model, whereas Method 2 also incorporates the “medium” and “high” model’s contribution, potentially improving performance if it aligns better with the EEG pattern.

Overall, Method 2 enhances robustness in uncertain affective conditions and achieves improved average accuracy and reduced inter-subject variability.

Method 3 represents the conventional decoding approach that does not incorporate the frustration discrimination model. In this setting, affective variability is regarded as noise, and the classifier is trained without considering emotional context. Specifically, the aBCI data from low, medium, and high frustration conditions were randomly sampled and combined as the training set. During inference, as described in Eq. (6), the input EEG segment $\textbf{X}$ is directly passed to the common classifier, and the final motor imagery (MI) prediction is obtained as:

6 $$\begin{aligned} \hat{y} = \arg \max \left( \mathcal {M}_{\text {all}}(\textbf{X}) \right) , \end{aligned}$$

where $\hat{y}$ denotes the predicted MI class (e.g., left or right hand).

To further examine the impact of training strategies, we considered two variants: **Method 3-1** employs all aBCI data pooled across frustration levels for training the state-agnostic decoder. **Method 3-2**, by contrast, uses only the non-frustration data collected during the initial stage as the training set, serving as a baseline condition.

For all variants, models were trained using the EEG segments corresponding to the full duration of participants’ MI execution. Each trial was segmented with a window length of 3000 sampling points and a step size of 100. Training and test sets were separated prior to any data augmentation to prevent data leakage. Model evaluation was performed using five-fold cross-validation to ensure robustness and reliability of the reported results.

## Data Availability

A sample of anonymized data and the code used in this study are available in the following GitHub repository: https://github.com/Delusionx1/aBCI_pBCI. For access to the full dataset, please contact the corresponding author via email at D.Zhang@bath.ac.uk. Access will be granted upon reasonable request and verification of intended data usage.
